# Priming with DNA encoding functional trimers and boosting with soluble trimeric protein elicited tier 2 neutralizing antibodies in non-human primates

**DOI:** 10.1186/1742-4690-9-S2-P314

**Published:** 2012-09-13

**Authors:** BK Chakrabarti, F Yu, K McKee, C Labranche, D Montefiori, JR Mascola, RT Wyatt

**Affiliations:** 1 International AIDS Vaccine Initiative/The Scripps Research Institute, La Jolla, CA, USA; 2 Vaccine Research Center/NIAID/NIH, Bethesda, MD, USA; 3 Duke University, Durham, NC, USA

## Background

All known HIV-1 gp120-directed broadly neutralizing antibodies efficiently recognize fully cleaved JR-FL spikes, however, the non-neutralizing gp120-directed antibodies only recognize the uncleaved JR-FL spikes. Therefore, as an immunogen, cleaved, functional spikes may selectively present neutralizing epitopes to B cells, perhaps more efficiently eliciting neutralizing antibodies. However, attempts to make soluble versions of Env that fully mimic the viral spike are yet to be successful. In vitro, this plasmid encodes for fully cleaved, cell-surface, trimeric JR-FL Env spikes, but only when expressed from a mini-LTR in a non-codon optimized state. In vivo, the LTR-driven, non-codon-optimized Env plasmid DNA, likely requires tat co-transfection in trans, and rev expression in cis, for efficient Env expression.

## Methods

To present functional, cleaved spikes to the immune system, we inoculated non-human primates (NHPs) with non-codon optimized JR-FL Env expressing plasmid DNA along with plasmid expressing Tat protein by electroporation. DNA priming was followed by boosts with soluble JR-FL gp140 trimers in adjuvant. As controls codon-optimized and CMV-driven Env plasmids were used to prime and three animals received protein in adjuvant only.

## Results

We report that the DNA priming elicited reasonable ELISA binding titers in NHPs and that the elicitation of neutralizing antibodies against Tier 1 viruses. Following trimer protein boosting of the DNA-primed animals, reasonable titers of Tier 2 neutralizing antibodies in the A3R5 assay were elicited. For the Tier 2 isolates tested to date, much of the neutralizing activity in sera maps to the CD4 binding site.

## Conclusion

We conclude that DNA priming by electroporation is an interesting and means to present functional, cleaved HIV-1 Env spikes to the B cell immune system to prime or initiate functional trimer-elicited neutralizing antibodies in primates.

